# Impact of pharmacy channel on adherence to oral oncolytics

**DOI:** 10.1186/s12913-017-2373-2

**Published:** 2017-06-19

**Authors:** Michael Stokes, Carolina Reyes, Yu Xia, Veronica Alas, Hans-Peter Goertz, Luke Boulanger

**Affiliations:** 1 Evidera, Montreal, QC Canada; 2 Genentech, San Francisco, CA USA; 3 Evidera, Lexington, MA USA; 4 GNS Healthcare, Cambridge, MA USA; 5 Truven Health Analytics, Cambridge, MA USA

**Keywords:** Oral oncolytics, Abandonment, Adherence, Discontinuation, Specialty pharmacy

## Abstract

**Background:**

Oral chemotherapy is increasingly prescribed to treat cancer. Despite its benefits, concerns have been raised regarding adherence to therapy. The study objective was to compare and measure adherence, persistence, and abandonment in patients filling prescriptions in traditional retail (TR) versus specialty pharmacy (SP) channels.

**Methods:**

Using a retrospective cohort design, we selected newly treated patients aged ≥18 years with a prescription for erlotinib, capecitabine, or imatinib during 2007–2011 from a Medco population of both United States commercial and Medicare health plans. Patients were classified according to pharmacy channel providing the medication. Abandonment was defined as a reversal following initial approval of the index prescription claim with no additional paid claims for agent within 90 days of reversal. Patients were considered adherent if the proportion of days covered between the date of the first and last oral prescription was ≥80%.

**Results:**

In our retrospective cohort, 11,972 filled their prescriptions within the SP channel, and 30,394 filled their prescriptions within the TR channels, respectively. The SP channel had the highest proportion of adherent patients compared with TR (71.6% vs. 56.4%, *P <* .001). Abandonment of the initial prescription was low with overall rates of only 1.7%. In multivariate models controlling for demographic characteristics, index oncolytic, days of supply, and copay, SP channel (relative to TR) was significantly associated with lower rates of abandonment and increased adherence.

**Conclusions:**

Pharmacy channel may be influential on abandonment and adherence. Lower rates of abandonment and higher rates of adherence were observed among SP patients versus TR.

**Electronic supplementary material:**

The online version of this article (doi:10.1186/s12913-017-2373-2) contains supplementary material, which is available to authorized users.

## Background

Oral drug therapy use for patients with cancer has increased sharply over the last few years and this trend is expected to increase over the next several decades [1]. Experts estimate that oral medications comprise 25-35% of the total cancer drugs in development [2–4]. In 2016, a report from IMS concluded that approximately 40% of total U.S. drug expenditures on targeted therapies were for oral formulations, up from 26% in 2010 [5].

Oral therapy provides potential advantages to patients. Oral agents may have different side effect profiles than their intravenous (IV) counterparts and patients may tolerate them better [6]. Patients often prefer oral agents to IV therapy because they offer more convenience as treatment interferes less with work and social activities, eliminating the need to travel to and from infusion clinics [7, 8]. As oncologists become more sensitive to patient preferences and quality of life, treatment options that offer more convenience and flexibility are likely to be used more often [9].

Despite the benefits of oral therapy, concerns have been raised regarding the risks of nonadherence as the responsibility of managing regimens and monitoring toxicities are centered more directly on the patient [1, 2, 10, 11]. One concern is under-dosing as a result of either nonadherence or dose reductions [12]. Under-dosing may lower drug plasma levels, thereby increasing the risk of cancer relapse or progression [13, 14]. Nonadherence may even cause clinicians to incorrectly attribute a patient’s poor response to an absence of drug activity, which may lead to unnecessary diagnostic tests, hospitalizations, and changes in the dose or regimen [9, 15]. Nonadherence is associated with an increased use of medical services [16].

Prior research has produced important findings regarding factors which adversely affect adherence in patients receiving oral oncolytics, including a lack of understanding of proper treatment administration, older age, complex dosing regimens, increased drug costs, and side effects [17–19]. However, there is limited data concerning the benefits of customized therapy management programs and whether specialty pharmacies can play a key role by working with physicians to ensure adherence and persistence in cancer patients. The purpose of this study was to examine this issue using a sample of patients newly treated with common oral oncolytics including erlotinib, capecitabine, and imatinib from the Medco research database. Erlotinib is approved for advanced non-small cell lung cancer (NSCLC) and advanced pancreatic cancer. Imatinib is approved for adult cancer indications including Philadelphia positive (Ph+) chronic myeloid leukemia, Ph + acute lymphoblastic leukemia, and unresectable/metastatic gastrointestinal stromal tumors. Capecitabine is indicated for colorectal and metastatic breast cancers.

## Methods

### Data source

A retrospective cohort analysis using the Medco research database was conducted to estimate the impact of the pharmacy channel on adherence and persistence in patients receiving oral oncolytics. Data were collected by Medco Health Solutions, formerly a large pharmacy benefits manager in the United States (US). As of 2011, the database included pharmacy claims from US commercial and Medicare health plans for over 60 million covered lives. Dates of health plan eligibility and demographic information were obtained from the eligibility files. The pharmacy claims included information on generic and brand medication names, national drug code (NDC), prescription fill dates, pharmacy channel, claim status (paid/reversed), as well as quantity and days supplied. Patient and provider information contained within the Medco database are de-identified, making it compliant with the Health Insurance Portability and Accountability Act privacy regulations.

### Patient eligibility criteria

The study sample included patients ≥18 years old with a prescription claim for erlotinib, imatinib, or capecitabine between July 1, 2007 and September 30, 2011. These agents were chosen because they are widely used to treat cancer and were commonly observed in the Medco database. Patients were classified into treatment groups (erlotinib, imatinib, or capecitabine) based on the first study oral oncolytic observed (i.e., the index oral oncolytic) during the study period. Only those without claims for the index oncolytic during the 6-month period prior to the index date (baseline period) were included to identify newly treated patients. The index date was defined as the fill date of the first oncolytic prescribed during the study period. Study measures were assessed over a variable follow-up period starting from the index date and ending at the date of disenrollment of pharmacy benefits or December 31, 2011 (last day of available data), whichever occurred earlier.

### Pharmacy channel groups

Patients were categorized into groups based on the pharmacy channel where they filled their index oncolytic prescription—Specialty (SP) or Traditional Retail (TR). Within the SP channel, pharmacists and nurses provide various customized therapy management services to patients aimed at encouraging adherence and adverse event monitoring. The TR channel consisted of a network of nearly 60,000 participating pharmacies throughout the US. Both the TR and SP pharmacy channels allowed for prescriptions with a daily supply >30 days.

### Abandonment, adherence, and persistence study outcomes

Abandonment occurs when a patient brings a prescription to the pharmacy and the claim is denied by the insurance provider either due to a Prior Authorization or Step Therapy requirement. Abandonment of the index prescription in this study was defined when the adjudication status of the first oncolytic prescription claim was reversed and there were no additional paid claims for the oncolytic within 90 days of the reversal. Patients were classified as having an “initial challenge but overcome” if their first fill for the oral oncolytic was reversed (because the claim was initially denied by their insurance) and they were able to fill a subsequent prescription (claim status = paid) for the same drug within 90 days of the initial reversal. Patients were classified as “approved without challenges” if the status of their index oncolytic was paid. Patients with missing data were classified as “unknown”.

Adherence to the index oral oncolytic was measured as the total days supply of all prescription fills between the first and last fill (days supply was capped at last fill date) divided by the number of days between the first and last fill (e.g., proportion of days covered). Values ≥80% were used as the cutoff value for defining adherence [20, 21]. This definition excluded patients who had one prescription fill or abandoned their index oncolytic prescription.

Persistence was defined as the duration of continuous use of the study drugs and allowed for brief gaps (<60 days) between the run-out date (fill date plus days supplied) of the prescription claim and the subsequent refill. Discontinuation was defined as an interruption in therapy ≥60 days between the run-out date (fill date plus days supplied) of the prescription claim and the subsequent refill. The persistence definition excluded patients who abandoned their index oncolytic. Pharmacy claims with status as reversed were excluded from the calculation of adherence and persistence; paid claims only were assessed. For instances where patients filled a prescription prior to the run-out date of their previous fill, we assumed patients would finish the current prescription before starting the refill.

### Data analyses

Primary data analyses were stratified according to pharmacy channel and included patients receiving erlotinib, imatinib, or capecitabine. Baseline demographic characteristics, including age, gender, geographical region, index study year and copay amount were summarized using descriptive statistics. For each patient, copay (per 30 day supply) was calculated by summing the copay amounts on prescription claims from the study index date until the end of follow-up and then dividing by the number of 30 day periods of total daily prescription supply dispensed. Copay was inflated to 2011 US dollars using the consumer price index. Copay is a fixed amount plan members pay out-of-pocket when filling prescriptions; this amount does not include other payments such as deductibles or coinsurance. Comparisons of baseline characteristics, adherence, abandonment, and persistence were assessed using *t*-tests for continuous variables and chi-square tests for categorical variables. Analyses examining the relationships between pharmacy channel and adherence and persistence were conducted according to index oncolytic received. We also estimated adherence and persistence measures among a subset of erlotinib patients with a pancreatic or lung cancer diagnosis recorded in the medical claims and for a subset of capecitabine patients with a breast or colorectal cancer diagnosis. As medical claims were only available for a subset of patients included in the study, these analyses were performed to confirm overall results in patients with a cancer diagnosis indicated for treatment with one of the oncolytics examined. Small sample sizes precluded analyses in imatinib patients. Finally, we examined adherence and persistence in a subset of patients who did not switch pharmacy channels during follow-up.

Logistic regression models were used to assess the impact of pharmacy channel on abandonment of the initial oncolytic prescription (yes vs. no [approved without challenges, initial challenge but overcome, and unknown groups]) and adherence (proportion of days covered: ≥80% vs. <80%). The adherence (proportion of days covered) model included age, gender, geographic region of residence, pharmacy channel (SP vs. TR), index oncolytic, average days of supply, and copay as covariates. Days of supply was included in the adherence model to rule-out the possibility that differences in adherence between SP and TR groups was simply related to differences in prescription days of supply. Since prescriptions with longer days of supply were thought only to affect abandonment of the initial oncolytic through higher copays, days of supply was omitted from the abandonment model. Statistical significance was evaluated at alpha = 0.05. SAS, version 9.2 (SAS Institute Inc., Cary, NC) was used for all analyses.

## Results

### Patient and pharmacy channel characteristics

Table 1 presents baseline characteristics results stratified by pharmacy channel where patients filled their index oncolytic. From an initial sample of 46,533 patients with a prescription for erlotinib, capecitabine or imatinib during July 1, 2007 through September 30, 2011, 42,366 were included in this study after exclusion criteria were applied (age < 18 years as of index date or prescription for the index oncolytic in the 6-month period prior to index). Among those included, 11,972 (28.3%) were included in the SP cohort and 30,394 (71.7%) in the TR cohort. SP patients were slightly younger compared with TR (mean age 63.9 vs. 64.4 years, *P* < 0.001). There was a slightly higher proportion of males in SP channel compared with TR (45.2% vs. 43.0%, *P* < 0.001). The distribution of patients residing in different geographical regions was also different across pharmacy channels (*P* < 0.001). A higher proportion of SP patients resided in the northeast (29.5%) versus TR (21.2%). There was also significant variation in the frequency of patients receiving a particular index oncolytic across pharmacy channel. The average copay among SP patients was ~$35 lower than TR ($221 vs. $256, *P* < 0.001). Similar relationships were observed in the subsets of patients included in analyses of abandonment and persistence (data not shown).

**Table 1:** Characteristics of patients according to pharmacy channel where index oral oncolytic prescription was filled^a^

Characteristic^b^	Specialty		Traditional Retail		*P*-value
Number of Patients	11,972		30,394		
Row Percent	28.3%		71.7%		
Mean (SD) Age	63.9 (12.5)		64.4 (12.9)		<0.001
Age Group, N (%):					<0.001
18–34 years	178	1.5%	493	1.6%	
35–44 years	620	5.2%	1616	5.3%	
45–54 years	1895	15.8%	4710	15.5%	
55–64 years	3452	28.8%	8239	27.1%	
65–74 years	3032	25.3%	7662	25.2%	
75–84 years	2625	21.9%	6734	22.2%	
85+ years	170	1.4%	940	3.1%	
Male, N (%):	5416	45.2%	13,068	43.0%	<0.001
Region, N (%):					<0.001
Northeast	3531	29.5%	6439	21.2%	
Midwest	2054	17.2%	6217	20.5%	
South	4173	34.9%	10,960	36.1%	
West	1813	15.1%	5107	16.8%	
Unknown	401	3.3%	1671	5.5%	
Index Study Year, N (%)					<0.001
2007	1118	9.3%	2670	8.8%	
2008	2118	17.7%	5880	19.4%	
2009	2860	23.9%	8176	26.9%	
2010	3451	28.8%	8240	27.1%	
2011	2425	20.3%	5428	17.9%	
Index Oncolytic Received, N (%)					<0.001
Erlotinib	4774	39.9%	10,297	33.9%	
Capecitabine	4906	41.0%	15,156	49.9%	
Imatinib	2292	19.1%	4941	16.3%	
Copay^c^ Amount Categorized					<0.001
$0–49	7112	59.4%	16,921	55.7%	
$50–100	1572	13.1%	4127	13.6%	
$101–150	761	6.4%	1877	6.2%	
$151–200	350	2.9%	920	3.0%	
$201–250	257	2.2%	656	2.2%	
$251–350	199	1.7%	651	2.1%	
$351–500	268	2.2%	900	3.0%	
$501–1000	694	5.8%	2137	7.0%	
> $1000	759	6.3%	2205	7.3%	
Mean (SD) Copay^c^ Amount (per 30 days)	220.7 (548.2)		256.1 (595.31)		<0.001
P25	16.4		15.0		
Median	36.0		40.0		
P75	111.4		142.86		
Mean (SD) Days Supplied Per Prescription	33.7 (20.7)		25.0 (12.5)		<0.001
Median	30.0		26.4		

*Note*: Study index date refers to the first date that a patient attempted to fill the index oral oncolytic prescription.

Abbreviations: SD, standard deviation; N, number of patients; P25, 25th percentile; P75, 75th percentile

^a^Pharmacy channel defined based on the channel where patient filled their initial (index) oral oncolytic

^b^Group comparisons were made using 2-sided Pearson chi-square for categorical measures, *t*-test statistic for mean age and Wilcoxon test for mean copay, *P*-values are presented for comparisons using traditional retail as reference group

^c^Mean copay amount (per 30 day supply) calculated using patients’ prescription drug claims for index oral oncolytic from study index until end of follow-up and inflated to 2011 US dollars

Approximately 50% of the oral oncolytic prescriptions filled in the SP channel were classified as mail order and 50% were classified as brick-and-mortar retail locations. Seventy-nine percent of SP patients and 91% of TR filled prescriptions with average daily supplies ≤30 days.

### Abandonment, adherence and persistence analyses

Rates of abandonment of the initial prescription were higher among TR compared with SP (Table 2, 2.0% vs. 0.8%, *P <* 0.001). A higher proportion of SP patients versus TR also had their index oncolytic approved without challenges (Table 2, 98.0% vs. 94.7%, *P <* 0.001).

**Table 2:** Unadjusted measures of abandonment, adherence and persistence according to pharmacy channel

Characteristic^a^	Specialty		Traditional Retail		*P*-value
Number of Patients	11,972		30,394		
Abandonment of Index Prescription					<0.001
Approved Without Challenges, N (%)	11,731	98.0%	28,788	94.7%	
Initial Challenge but Overcome, N (%)	118	1.0%	875	2.9%	
Initial Challenge Unknown if Overcome, N (%)^b^	3	0.0%	43	0.1%	
Abandoned, N (%)^c^	97	0.8%	604	2.0%	
Unknown, N (%)^d^	23	0.2%	84	0.3%	
Adherence - Proportion of Days Covered between the First and Last Fill^e^					
Mean (SD)	0.86 (0.2)		0.79 (0.2)		<0.001
Number with >1 fill	9194		22,219		
Adherent, N (%)	6586	71.6%	12,542	56.4%	<0.001
Adherence - Proportion Days Covered between First and Last Fill, N (%)					<0.001
0–0.20	12	0.1%	246	1.1%	
0.21–0.40	241	2.6%	1273	5.7%	
0.41–0.60	984	10.7%	3815	17.2%	
0.61–0.70	733	8.0%	2571	11.6%	
0.70–0.79	638	6.9%	1772	8.0%	
0.80–0.90	965	10.5%	2182	9.8%	
0.90–1.00	5621	61.1%	10,360	46.6%	
Persistence^f,g^ - Time until Discontinuation of Index Oncolytic (days)					
Number Filling Prescription for Index Oncolytic	11,849		29,663		
Mean (SD)	186.2 (235.2)		161.8 (218.5)		<0.001
Median	94		83		
Minimum–Maximum	0–1622		0–1613		

*Abbreviations*: *SD* standard deviation, *N* number of patients

^a^Group comparisons were made using 2-sided Pearson chi-square for categorical measures and *t*-test statistics for continuous measures, *P*-values are presented for comparisons using traditional retail as reference group

^b^Unable to determine if hurdle was overcome because follow-up time was censored

^c^Abandonment measure refers to whether the patient was able to successfully fill prescription within 90 days following an initial challenge (if no, prescription was considered abandoned)

^d^No information provided regarding the patient’s first fill status, could not determine if prescription was filled

^e^Adherence definition excluded patients who only had one prescription fill or abandoned their index prescription (calculation included *n* = 9194 and *n* = 22,219 specialty and traditional retail patients, respectively)

^f^Time until discontinuation (in days) of index oral oncolytic, allowing for a 60-day gap in therapy between the run-out date of the medication and the subsequent fill

^g^The definition of persistence excluded patients who abandoned their index oncolytic (calculation included *n* = 11,849 and *n* = 29,663 specialty and traditional retail patients, respectively)

A larger proportion of SP channel patients were more adherent (proportion of days covered ≥80%) to their index oncolytic compared with TR (71.6% vs. 56.4%, *P <* 0.001) (Table 2). The average proportion of days covered was also significantly higher for SP (0.86) versus TR (0.79, *P <* 0.001) (Table 2).

SP patients remained on their index oral oncolytic an average of 24.4 days longer versus TR (186.2 vs. 161.8 days, *P <* 0.001) (Table 2).

Similar relationships were observed when the data were stratified according to index oncolytic received (Table 3) and in analyses examining the subset of patients with a cancer diagnosis recorded in the medical claims (Additional file 1) as well as the subset that did not switch pharmacy channels (Additional file 2). The results presented in Tables 2 and 3, Additional file 1, and Additional file 2 are not adjusted for differences in baseline characteristics.

**Table 3:** Unadjusted measures of abandonment, adherence and persistence, by index oral oncolytic received and pharmacy channel

	Erlotinib				Imatinib				Capecitabine			
Characteristic^a^	Specialty		Traditional Retail		Specialty		Traditional Retail		Specialty		Traditional Retail	
Number Prescribed Index Oncolytic	4774		10,297		2292		4941		4906		15,156	
Abandonment of Index Prescription	^f^				^f^				^f^			
Approved Without Challenges, N (%)	4643	97.3%	9644	93.7%	2271	99.1%	4688	94.9%	4817	98.2%	14,456	95.4%
Initial Challenge but Overcome, N (%)	66	1.4%	358	3.5%	7	0.3%	157	3.2%	45	0.9%	360	2.4%
Initial Challenge Unknown if Overcome, N (%)^b^	3	0.1%	25	0.2%	0	0.0%	3	0.1%	0	0.0%	15	0.1%
Abandoned, N (%)^c^	50	1.0%	239	2.3%	11	0.5%	79	1.6%	36	0.7%	286	1.9%
Unknown, N (%)^d^	12	0.3%	31	0.3%	3	0.1%	14	0.3%	8	0.2%	39	0.3%
Adherence - Proportion of Days Covered between the First and Last Fill												
MPR Mean (SD)	92.1 (14.0)^f^		89.6 (17.1)		86.8 (18.9)^f^		82.5 (21.2)		80.7 (20.4)^f^		70.6 (22.5)	
Number with >1 Fill	3439		7091		2046		4230		3709		10,898	
Adherent, N (%)	2888	84.0%^f^	5691	80.3%	1527	74.6%^f^	2813	66.5%	2171	58.5%^f^	4038	37.1%
Persistence^e^ - Time until Discontinuation of Index Oncolytic (days)												
Number Filling Prescription for Index Oncolytic	4709		10,002		2278		4845		4862		14,816	
Mean (SD)	146.0 (166.7)^f^		135.8 (169.9)		423.2 (359.0)^f^		394.5 (354.9)		114.2 (120.2)^f^		103.2 (115.3)	
Median	89		79		324		292		78		63	
Minimum–Maximum	0–1441		0–1613		0–1622		0–1601		0–1316		0–1397	

*Note*: NDC codes 50242006201, 50242006301, 50242006401, 54569584700, 54569584800, 54868529000, 54868544700, 54868547400 used to identify erlotinib, NDC codes 00004110020, 00004110051, 00004110116, 00004110150, 54569571700, 54868414300-54868414303, 54868526000-54868526009 used to identify capecitabine, NDC codes 00078037366, 00078040105, 00078040134, 00078040215, 00078043815, 54569584600, 54868528900-54868528904, 54868542700-54868542703 used to identify imatinib

*Abbreviations*: *SD* standard deviation, *N* number of patients

^a^Group comparisons were made using 2-sided Pearson chi-square for categorical measures and *t*-test statistics for continuous measures within each index oncolytic population, *p*-values are presented for comparisons using traditional retail as reference group

^b^Unable to determine if hurdle overcome because data were censored

^c^Abandonment measure refers to whether patient was able to successfully fill prescription within 90 days following an initial challenge (if no, prescription was considered abandoned)

^d^No information provided regarding the patients first fill status, cannot determine if prescription was filled

^e^Time until discontinuation (in days) of index oral oncolytic, allowing for a 60 day gap in therapy between the run-out date of the medication and the subsequent fill

^f^ *P* < 0.001

### Multivariate analyses

Table 4 presents results of the multivariate model for abandonment of the initial oncolytic prescription. After covariate adjustment, SP channel was associated with significantly lower rates of abandonment (odds ratio, OR [95% confidence interval, CI], 0.44 [0.35–0.55]). Higher copay amounts were associated with a higher likelihood of abandonment.

**Table 4:** Logistic regression model: abandonment of initial oncolytic prescription

Determinant	Odds Ratio	95% CI
Age, Reference <65 years		
≥ 65 years	1.25	1.04–1.51
Gender, Reference Female		
Male	1.16	0.99–1.36
Geographic Region, Reference South		
Northeast	0.64	0.51–0.81
Midwest	1.10	0.90–1.35
West	1.00	0.78–1.27
Other/missing	1.48	1.01–2.17
Population, Reference Retail Pharmacy		
Specialty	0.44	0.35–0.55
Copay, Reference $0–49		
$50–100	1.55	1.14–2.12
$101–150	1.55	1.02–2.35
$151–200	1.94	1.17–3.24
$201–250	3.43	2.15–5.47
$251–350	4.54	3.00–6.93
$351–500	5.22	3.65–7.46
$501–1000	4.89	3.71–6.44
> $1000	13.69	10.87–17.23
Index Oncolytic, Reference Erlotinib		
Capecitabine	1.41	1.15-1.74
Imatinib	0.94	0.73-1.22

*Abbreviations*: *CI* confidence interval

Table 5 displays multivariate results for adherence (proportion of days covered ≥80%) to the index oncolytic. After covariate adjustment, SP channel was associated with a higher level of adherence compared with TR (Odds ratio, OR [95% CI], 1.99 [1.87–2.11]). Higher copays were associated with lower adherence. The average prescription days of supply category ≥90 days was associated with better adherence.

**Table 5:** Logistic regression model: adherence to index oral oncolytic^a^

Determinant	Odds Ratio	95% CI
Age, Reference <65 Years		
≥ 65 years	1.04	0.98–1.09
Gender, Reference Female		
Male	1.15	1.09–1.21
Geographic Region, Reference South		
Northeast	0.93	0.87–0.99
Midwest	1.12	1.04–1.20
West	0.97	0.90–1.05
Unknown	1.02	0.87–1.18
Population, Reference Retail Pharmacy		
Specialty	1.99	1.87–2.11
Copay, Reference $0–49		
$50–100	0.74	0.69–0.80
$101–150	0.83	0.75–0.92
$151–200	0.74	0.65–0.86
$201–250	0.65	0.55–0.77
$251–350	0.89	0.75–1.06
$351–500	0.93	0.80–1.09
$501–1000	0.92	0.83–1.02
> $1000	0.74	0.65–0.83
Prescription Days of Supply, Reference ≤30		
31-89	0.28	0.26-0.30
90+	1.66	1.35-2.03
Index Oncolytic, Reference Erlotinib		
Capecitabine	0.16	0.15-0.17
Imatinib	0.53	0.49-0.58

*Note*: patients assumed to be adherent if proportion of days covered ≥80%, Mean p[adherence] for SP = 0.76, Mean p[adherence] for TR = 0.59, Mean p[adherence] for patients receiving erlotinib = 0.83, Mean p[adherence] for patients receiving capecitabine = 0.42, Mean p[adherence] for patients receiving imatinib = 0.70

*Abbreviations*: *CI* confidence interval, P[adherence], predicted probability of adherence

^a^Based on the proportion of days covered between first and last fill of index oncolytic, analysis includes patients who filled their index oncolytic >1 time as adherence could only be measured for these patients

## Discussion

We used data from the Medco research database to estimate the impact of pharmacy channel on adherence and persistence among patients receiving erlotinib, imatinib, or capecitabine during 2007–2011. Our results indicate that filling prescriptions in a SP channel may lead to greater adherence compared with TR. This finding is consistent with previous research examining SPs [22–25]. In addition, SP patients were less likely to abandon their initial prescription and remained on treatment longer versus TR. Prior studies have focused on adherence in cancer patients receiving oral therapies [11, 13, 26–31]. However, there are limited data examining the impact of SP channel on adherence. The overall mean proportion of days covered (adherence) in the current study was 79% for the TR channel (90%, 83%, and 71% for TR patients receiving erlotinib, imatinib, and capecitabine, respectively) and 86% for SP (92%, 87%, and 81% for SP patients receiving erlotinib, imatinib, and capecitabine, respectively). Prior retrospective studies examining adherence to imatinib and utilizing prescription claims data have reported mean medication possession ratios between 73%–90% [28, 29, 31]. Mean MPRs and adherence rates for imatinib estimated as part of this current study were within the reported range of these prior studies. Among studies of adherence to breast cancer medications utilizing prescription refills, adherence rates (proportion of days covered ≥80%) over 1–2 years varied significantly, from 62%–89% [32]. Many published rates in breast cancer are based on studies assessing adherence to tamoxifen regimens, which may differ significantly from typically shorter oral chemotherapy regimens [26, 32]. A few studies assessing capecitabine adherence in patients with breast, colorectal, and GIST tumors were identified [11, 30, 33, 34]. One study was a randomized controlled trial of patients with breast cancer where adherence was calculated as the number of doses taken (as measured by a microelectronic monitoring system) divided by the number of planned doses. Average adherence of 78% was reported across all cycles [34]. Other studies assessing capecitabine used patient self-reports and noted adherence rates of 76.7% [11] and 91% [30] which were higher relative to the current study. A possible explanation for this is that Bhattacharya et al. and Winterhalder et al. relied on patient self-reports, which have a tendency to overstate compliance. In addition, the retrospective database design of the current study allowed for an observational period spanning multiple years whereas these prior studies examining capecitabine had follow-up periods of only 4–6 months [30]. The study by Walter et al., reported capecitabine adherence rates of only 61% with microelectronic monitoring system, which was more in line with the current study, compared to 99% with patient self-report. Relatively few studies examining adherence to erlotinib were identified. One study from the Netherlands utilizing patient self-reports recorded a mean MPR of 96.8% for erlotinib [35] which was comparable to the mean MPRs of 90% + reported in this study. Rates of adherence may vary across different studies due to factors such as study design, cancer type, patients’ overall disease burden, or treatment received. As part of this study, we also performed analyses in a subset of erlotinib and capecitabine patients with a cancer diagnosis recorded in the medical claims. Among the erlotinib subset, 86% had lung cancer and 14% had pancreatic cancer. Forty-seven percent of the capecitabine subgroup had breast cancer and 53% had colorectal cancer. The mean proportion of days covered within both tumor and oral oncolytic subgroups were consistent with results of the full patient sample stratified only by oncolytic received.

Our results suggest that customized therapy management programs instituted at SPs can have a positive influence on adherence and persistence in cancer patients prescribed oral oncolytics. SP therapy management programs provide additional services to patients which are likely to contribute to improved levels of adherence compared with TR. For example, when patients start new prescriptions in the SP channel, pharmacists or nurses counsel patients by telephone, often verifying that the appropriate dose has been prescribed, explaining adverse effects, potential drug interactions, dosing requirements, instructions for use, and storage requirements. Patients might also be contacted prior to each refill to monitor outcomes and encourage adherence. Some SPs even offer 24-h nursing support for patients throughout their therapy as well as home visits prior to dispensing the medication. Many SPs also have therapeutic centers dedicated to oncology with staff specializing in this area.

The level of cost sharing was also a significant factor in determining adherence to oral medication. Patients with higher copays were more likely to have lower rates of adherence and higher rates of abandonment of the index oncolytic. Many prior studies have demonstrated a clear relationship between higher copays and decreased adherence [36–38]. Other unmeasured factors such as total household income or the proportion of out-of-pocket expenses in relation to income may have also played a role in affecting adherence. These variables were not available in the database.

This study used data from a healthcare claims database. Claims data are primarily collected for reimbursement purposes rather than research. The classification of patients into study groups and the measures derived from the data relied on inferences based upon the information appearing on the claims. Thus, there is the potential for coding inaccuracies leading to the misclassification of certain events or measures of interest. For example, the abandonment measure was inferred from the information appearing on the claims. Claim reversals were examined as an indication that the patient was having difficulty filling the prescription. The accuracy of our algorithm in identifying true instances of abandonment is not known. It is possible that some reversed claims were excluded from our dataset if the reversal occurred prior to the claims being loaded into the database. Consequentially, the abandonment rate might have been underestimated. Indeed, a prior study using nationally representative pharmacy data reported first fill abandonment rates of 6.2%, 13.5%, and 12.8% for capecitabine, imatinib, and erlotinib, respectively; rates which are higher compared to our study [39]. Finally, our adherence measure was not an actual measure of medication consumption. We assumed that patients actually took the medication after filling prescriptions.

There were differences observed in the baseline characteristics of patients filling prescriptions in the SP channel and those utilizing TR which could have influenced results. Therefore, multivariate analyses controlling for age, gender, geographic region of residence, index oncolytic, average days of supply, and copay were conducted to confirm unadjusted results showing that SP patients have higher rates of adherence (unadjusted OR = 1.95) and lower rates of abandonment (unadjusted OR = 0.40) compared with TR. In multivariate models, SP channel compared with TR was associated with significantly lower rates of abandonment (adjusted OR = 0.44) and higher rates of adherence (adjusted OR = 1.99) affirming the unadjusted results.

Our analyses were also limited by the variables available in the database. Data on overall income was not available. Hence, we could not explore the burden of patients’ out-of-pocket expenses relative to their overall income, which could have had a greater effect on adherence compared with their actual expense. We did not know whether patients were receiving assistance with copays from patient programs which could have also influenced results. A minimum post-eligibility period was not used for analyses to avoid bias from excluding patients with shorter follow-up periods due to death. We assumed death was the most likely reason for health coverage termination and that bias due to censored data would be minimal. Mortality data were not available to confirm this assumption. For most patients, we did not have access to their medical claims data. Therefore, it was inferred that patients were taking their oral chemotherapy to treat advanced cancer. An analysis was conducted to assess patient subgroups with medical claims containing a cancer diagnosis indicated for treatment with an oncolytic. Subgroup analyses were consistent with overall results. We also assumed differences in comorbidity or the severity of the patients’ cancer would be negligible across pharmacy channel cohorts and would not significantly influence abandonment or adherence metrics. Finally, it should be noted that some TR pharmacies also have adherence and specialty clinical programs; the degree to which these programs were implemented was not captured in the database. Therefore, it is difficult to determine what effect this may have had on results.

The rapid growth in the development of specialty pharmaceuticals including oral oncolytics has increasingly led to the transfer of responsibility for obtaining and administering complex and costly medications from healthcare providers and hospitals to patients in the community [40]. These changes have driven new models of care including the rise of customized therapy management programs with the aim of improving otherwise unfavorable medication use behaviors, mitigating waste, and improving outcomes [40]. The published evidence, although limited, does suggest that the majority of patient support and therapy management programs are having a positive impact [40].

## Conclusions

Patients filling prescriptions in the SP channel were more likely to achieve adherence compared with patients using TR. Pharmacy channel also appears to be influential on abandonment, with lower rates observed among SP patients compared with TR.

## Additional files

Additional file 1: Unadjusted measures of abandonment, adherence, and persistence: patient subset with cancer diagnosis code. (DOCX 15 kb)

Additional file 2: Unadjusted measures of abandonment, adherence, and persistence: patient subset who did not switch pharmacy channels. (DOCX 13 kb)

## Abbreviations

IV: Intravenous
NDC: National drug code
NSCLC: Non-small cell lung cancer
Ph +: Philadelphia positive
SP: Specialty pharmacy
TR: Traditional retail
US: United States
