# Supplementary material for: Impact of pharmacy channel on adherence to oral oncolytics
Source: BMC Health Serv Res. 2017 Jun 19;17:414. doi: 10.1186/s12913-017-2373-2 (PMC5477418; doi:10.1186/s12913-017-2373-2)
Supplement: Supplementary file 1 — Unadjusted measures of abandonment, adherence, and persistence: patient subset with cancer diagnosis code. (DOCX 15 kb) [file 12913_2017_2373_MOESM1_ESM.docx]

**Additional File 1**

**Unadjusted Measures of Abandonment, Adherence and Persistence: Patient Subset with Cancer Diagnosis Code**

|  | **Erlotinib^b^** | | | | **Capecitabine^c^** | | | |
| --- | --- | --- | --- | --- | --- | --- | --- | --- |
| **Characteristic^a^** | **Specialty** | | **Traditional Retail** | | **Specialty** | | **Traditional Retail** | |
| Number Prescribed Index Oncolytic | 703 | | 1,064 | | 778 | | 2,025 | |
| Abandonment of Index Prescription | ^h^ | |  |  | ^h^ | |  |  |
| Approved Without Challenges, N (%) | 692 | 98.4% | 1,020 | 95.8% | 760 | 97.7% | 1,924 | 95.0% |
| Initial Challenge but Overcome, N (%) | 4 | 0.6% | 25 | 2.3% | 13 | 1.7% | 48 | 2.4% |
| Initial Challenge Unknown if Overcome, N (%)^d^ | 0 | 0.0% | 2 | 0.2% | 0 | 0.0% | 0 | 0.0% |
| Abandoned, N (%)^e^ | 6 | 0.9% | 16 | 2.3% | 3 | 0.4% | 43 | 2.1% |
| Unknown, N (%)^f^ | 1 | 0.1% | 3 | 0.3% | 2 | 0.3% | 10 | 0.5% |
| Adherence - Proportion of Days Covered between the First and Last Fill |  |  |  |  |  |  |  |  |
| MPR Mean (SD) | 92.3 (14.1)^h^ | | 89.1 (17.8) | | 79.4 (19.7)^h^ | | 69.6 (22.0) | |
| Number with >1 Fill | 541 | | 749 | | 608 | | 1,491 | |
| Adherent, N (%) | 463 | 85.6%^h^ | 590 | 78.8% | 333 | 54.8%^h^ | 508 | 34.1% |
| Persistence^g^ - Time until Discontinuation of Index Oncolytic (days) |  | |  | |  |  |  |  |
| Number Filling Prescription for Index Oncolytic | 4,709 | | 10,002 | | 772 | | 14,816 | |
| Mean (SD) | 155.7 (175.9) | | 141.0 (179.0) | | 116.9 (111.7) | | 107.8 (117.3) | |
| Median | 91 | | 77.5 | | 85 | | 68 | |
| Minimum–Maximum | 23–1,420 | | 9–1,488 | | 6–824 | | 0–1,138 | |

^a^Group comparisons were made using 2-sided Pearson chi-square for categorical measures and *t*-test statistics for continuous measures within each index oncolytic population, *p*-values are presented for comparisons using traditional retail as reference group

^b^Subset of erlotinib patients with lung (ICD-9-CM 162.2-162.9, 163.x) or pancreatic cancer (ICD-9-CM 157.x) indications identified using medical claims data

^c^Subset of capecitabine patients with breast (ICD-9-CM 174.x, 175.x) or colorectal cancer (ICD-9-CM 154.0, 154.1, 153.x) indications identified using medical claims data

^d^Unable to determine if hurdle overcome because data were censored

^e^Abandonment measure refers to whether patient was able to successfully fill prescription within 90 days following an initial challenge (if no, prescription was considered abandoned)

^f^No information provided regarding the patients first fill status, cannot determine if prescription was filled

^g^Time until discontinuation (in days) of index oral oncolytic, allowing for a 60 day gap in therapy between the run-out date of the medication and the subsequent fill

^h^*P*<0.05
