# Supplementary material for: Impact of pharmacy channel on adherence to oral oncolytics
Source: BMC Health Serv Res. 2017 Jun 19;17:414. doi: 10.1186/s12913-017-2373-2 (PMC5477418; doi:10.1186/s12913-017-2373-2)
Supplement: Supplementary file 2 — Unadjusted measures of abandonment, adherence, and persistence: patient subset who did not switch pharmacy channels. (DOCX 13 kb) [file 12913_2017_2373_MOESM2_ESM.docx]

**Additional File 2**

**Unadjusted Measures of Abandonment, Adherence and Persistence: Patient Subset Who Did Not Switch Pharmacy Channels**

| **Characteristic^a^** | **Specialty** | | **Traditional Retail** | | ***P*-value** |
| --- | --- | --- | --- | --- | --- |
| Adherence - Proportion of Days Covered between the First and Last Fill^b^ |  |  |  |  |  |
| Number with >1 fill | 8,457 | | 17,452 | |  |
| Mean (SD) | 0.86 (0.2) | | 0.79 (0.2) | | <0.001 |
| Adherent, N (%) | 6,062 | 71.7% | 9,890 | 56.7% | <0.001 |
| Persistence^c,d^ - Time until Discontinuation of Index Oncolytic (days) |  | |  | |  |
| Number Filling Prescription for Index Oncolytic | 11,031 | | 24,288 | |  |
| Mean (SD) | 184.6 (233.8) | | 145.3 (202.9) | | <0.001 |
| Median | 92 | | 70 | |  |
| Minimum–Maximum | 0–1,622 | | 0–1,601 | |  |

^a^Group comparisons were made using 2-sided Pearson chi-square for categorical measures and *t*-test statistics for continuous measures , *P*-values are presented for comparisons using traditional retail as reference group

^b^Adherence definition excluded patients who only had one prescription fill or abandoned their index prescription

^c^Time until discontinuation (in days) of index oral oncolytic, allowing for a 60-day gap in therapy between the run-out date of the medication and the subsequent fill

^d^The definition of persistence excluded patients who abandoned their index oncolytic

Abbreviations: SD, standard deviation; N, number of patients
